# Ventrolateral Prefrontal Cortex Contributes to Human Motor Learning

**DOI:** 10.1523/ENEURO.0269-22.2022

**Published:** 2022-09-28

**Authors:** Neeraj Kumar, Ananda Sidarta, Chelsea Smith, David J. Ostry

**Affiliations:** 1 Department of Psychology, McGill University, Montreal, Quebec H3A1G1, Canada; 2 Department of Liberal Arts, Indian Institute of Technology Hyderabad, Hyderabad, Telangana 502285, India; 3 Rehabilitation Research Institute of Singapore, Nanyang Technological University, Singapore 308232; 4 Haskins Laboratories, New Haven, CT 06511

**Keywords:** motor learning, reinforcement, TMS

## Abstract

This study assesses the involvement in human motor learning, of the ventrolateral prefrontal cortex (BA 9/46v), a somatic region in the middle frontal gyrus. The potential involvement of this cortical area in motor learning is suggested by studies in nonhuman primates which have found anatomic connections between this area and sensorimotor regions in frontal and parietal cortex, and also with basal ganglia output zones. It is likewise suggested by electrophysiological studies which have shown that activity in this region is implicated in somatic sensory memory and is also influenced by reward. We directly tested the hypothesis that area 9/46v is involved in reinforcement-based motor learning in humans. Participants performed reaching movements to a hidden target and received positive feedback when successful. Before the learning task, we applied continuous theta burst stimulation (cTBS) to disrupt activity in 9/46v in the left or right hemisphere. A control group received sham cTBS. The data showed that cTBS to left 9/46v almost entirely eliminated motor learning, whereas learning was not different from sham stimulation when cTBS was applied to the same zone in the right hemisphere. Additional analyses showed that the basic reward-history-dependent pattern of movements was preserved but more variable following left hemisphere stimulation, which suggests an overall deficit in somatic memory for target location or target directed movement rather than reward processing per se. The results indicate that area 9/46v is part of the human motor learning circuit.

## Significance Statement

Prefrontal cortex may contribute to motor learning as it is known to be involved in planning, executive control, and motivation or reward processing (Miller and Cohen, 2001). Here, we focused on ventrolateral prefrontal cortex (BA 9/46v), an area which has been shown to be linked neuroanatomically and electro-physiologically to sensorimotor regions of the brain and to circuits involved in reinforcement. Using continuous theta burst stimulation (cTBS) to this region before a reinforcement-based motor learning task, we found a significant reduction in learning. This suggests that this zone in the lateral prefrontal cortex contributes to motor learning which is mediated by reward.

## Introduction

The brain structures involved in human motor learning have been studied extensively. Areas in frontal and parietal cortex, cerebellum and basal ganglia have each been shown to contribute to learning and retention, although their weighting differs between tasks. In contrast, prefrontal cortex has received little attention to date in the context of motor learning (but see Anguera et al., 2010 and Codol et al., 2020) although regions within prefrontal cortex are known to be neuroanatomically connected to sensorimotor related regions of other structures which are implicated in learning. The present study focuses on ventrolateral prefrontal cortex (BA 9/46v), a somatic region in the middle frontal gyrus directly above the ascending anterior ramus of the lateral fissure that separates BA 44 and 45, and has neuroanatomical connections to premotor, somatosensory and basal ganglia structures. By disrupting this area using magnetic brain stimulation, we test for its participation in human motor learning.

The focus on 9/46v is motivated by both electrophysiological findings and neuroanatomical connectivity. Studies in nonhuman primates have identified a homologous somatic region in the inferior bank of the principal sulcus which is interconnected with areas PF and PFG in the inferior parietal lobe (or supramarginal gyrus in humans) and second somatosensory cortex in the parietal operculum (Preuss and Goldman‐Rakic, 1989; Petrides and Pandya, 2002; for a summary, see Yeterian et al., 2012). This area also communicates with the hand area of ventral premotor cortex and likewise receives inputs from globus pallidus and substantia nigra of the basal ganglia (Middleton and Strick, 2002). In electrophysiological studies, this same region has been implicated in somatic sensory memory and decision-making (Romo et al., 1999).

We tested for the involvement of 9/46v using a reinforcement learning task. In reinforcement-based motor learning, positive feedback provides behavioral reinforcement, inducing plasticity in motor, somatic and reward-related networks (Bernardi et al., 2015; Sidarta et al., 2016). The involvement of the middle frontal gyrus in reinforced sequence learning has been demonstrated using repetitive TMS (Dayan et al., 2018). Area 9/46v involvement in both reinforcement learning (Fermin et al., 2016) and visuomotor adaptation (Anguera et al., 2010) has been observed in studies using fMRI. Other parts of the prefrontal cortex, in particular, ventromedial prefrontal cortex and orbitofrontal cortex have been implicated in reward-based learning more generally. In the nonhuman primate literature, activity in dorsolateral prefrontal cortex during a delay period was found to be related to the amount of reward received and the type of responses to be performed (Hikosaka and Watanabe, 2000; Wallis and Miller, 2003). Moreover, there is evidence that the lateral prefrontal cortex carries reciprocal projections with the midbrain dopaminergic neurons (Williams and Goldman-Rakic, 1998; Frankle et al., 2006), as well as with the orbitofrontal cortex (Barbas and Pandya, 1989).

Participants in the present study were assigned to one of three experimental conditions in which continuous theta-burst transcranial magnetic stimulation (cTBS) was applied to either left or right 9/46v, with the goal of disrupting activity in the target zone, or to a sham stimulation group. This was followed by a motor learning task in which participants performed reaching movements to a hidden target. The participants were given positive feedback when the movement was successful, that is, when it had landed in the target zone. We found that disruption of area 9/46v before learning had a detrimental effect on both learning rate and on the overall number of successful (and thus rewarded) movements. This is consistent with its participation in reinforcement-based motor learning.

## Materials and Methods

### Participants

Fifty-four healthy right-handed young adults (19 men, 35 women) were recruited and randomly assigned into either a left hemisphere (left 9/46v, *N* = 18), right hemisphere (right 9/46v, *N* = 18), or sham stimulation condition (sham, *N* = 18). Handedness was assessed using the Edinburgh handedness inventory (Oldfield, 1971). All procedures were approved by the McGill University Faculty of Medicine Institutional Review Board and participants provided written informed consent.

### Experimental design

**Figure 1.**
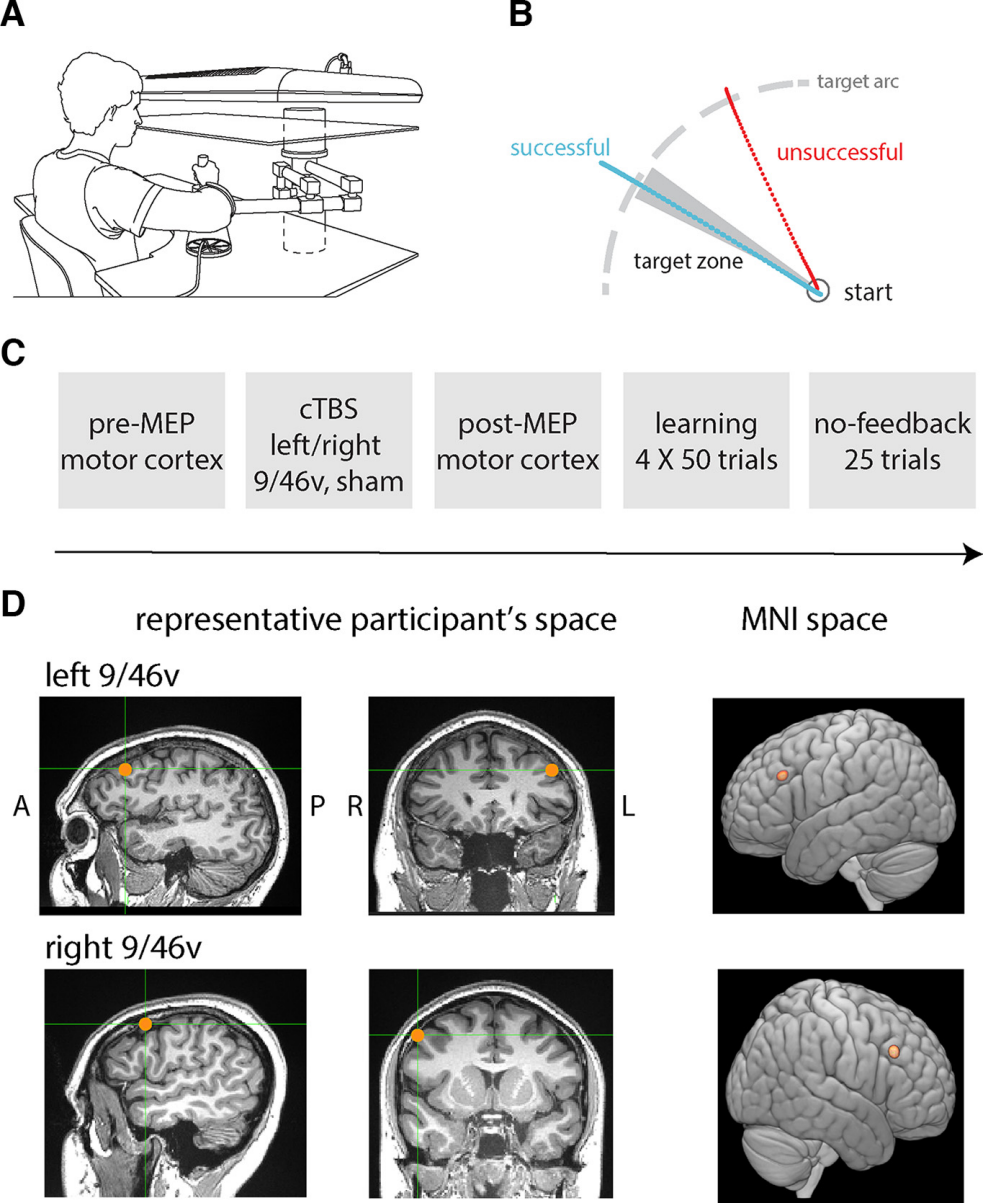
Participants learned to make movements to a hidden target, and positive feedback was provided for successful movements. ***A***, Participants made movements holding a robotic manipulandum. ***B***, Schematic of the task. Participants made outward movements. If the movement direction fell within the hidden target zone, positive feedback was provided to indicate success. No feedback was given in the case of an unsuccessful movement. ***C***, Experimental sequence. MEPs were elicited from the motor hot-spot in the left or right hemisphere before stimulation (cTBS to right or left 9/46v or sham stimulation). MEPs were again recorded 10 min after stimulation followed by the motor learning trials. In the no-feedback session at the end, participants were not provided with feedback on the success of the movement. ***D***, Location of the stimulation site in representative participants from the left 9/46v and right 9/46v condition, shown in the sagittal (right panel) and coronal (middle panel) planes. The average location of the stimulation site (red circle) across participants in the MNI brain.

Participants held a vertical handle attached to the end of a 2-df robotic manipulandum (Interactive Motion Technologies). They were seated with their right shoulder abducted to ∼70° and the elbow supported by an air sled. A semi-silvered mirror, which served as a display screen, was placed just below eye level and blocked the vision of the arm and the robot handle (Fig. 1*A*). A white start circle, 20 mm in diameter, was positioned on the display screen ∼30 cm in front of the participant, on the body midline. A 1-cm white arc was shown on the left of the screen during familiarization trials (Fig. 1*B*). During the familiarization phase, participants were instructed to move to any point on the arc after the “Go” cue appeared and to make straight movements without corrections. A cursor, which represented the instantaneous handle position in space, was removed once the arm moved outside of the white start circle. The required movement duration was 500–700 ms, but there was no penalty if the movement did not end on time or outside the target arc. Once the movement ended, the robot brought the arm back to the start position.

Following the familiarization training, the target arc was removed. The participant was instructed to move toward the now hidden arc and was told there was a target located in the arc. Then, each participant made 15 movements without receiving feedback of any kind. A target direction was then set for each subject separately to correspond to the direction of the first movement after the 15th trial that fell between 110° and 160° (second quadrant at the left). Positive feedback (an animated explosion, a pleasant tone, and a score) was provided for this movement. Participants were told that their task was to repeat the same successful movement throughout the course of training. Positive feedback was dependent solely on movement direction at peak velocity although participants were provided feedback on distance for training purposes during familiarization trials. The width of the target zone was 5°, and positive feedback was provided if the angular deviation was within ±2.5° of the center line. The width and position of the reinforced direction were fixed. Altogether, the participants completed four blocks of 50 training trials with positive feedback when successful. This was followed by 25 further movement trials with no feedback. For these trials, participants were told to aim in the direction in which they had been rewarded previously. They were also told that no reward would be given even if they were accurate. The sequence of different phases of the experiment is shown in Figure 1*C*.

### Stimulation sites

Before the study, each participant underwent an MRI scan at the Montreal Neurologic Institute Brain Imaging Centre. Structural images were acquired with a T1-weighted 3D MPRAGE sequence as follows: TR = 2300 ms; TE = 2.98 ms; slices = 192; thickness = 1 mm (no gap); FA = 90°; and FOV = 256 × 256 mm, iPAT mode = ON (acceleration factor 2×).

The stimulation location in area 9/46v was identified for each subject separately, in the following manner. The identification starts with pars opercularis and pars triangularis in the inferior frontal gyrus, which are separated by the ascending anterior ramus of the lateral fissure (Petrides and Pandya, 2002). This ascending sulcus runs up from the lateral fissure and is almost perpendicular to the inferior frontal sulcus. The stimulation site, as shown in Figure 1*D*, lies in the middle frontal gyrus, medial to ascending anterior ramus of the lateral fissure and between two posterior middle frontal gyrus sulci, the posterior middle frontal sulcus (anterior) and posterior middle frontal sulcus (intermediate; Petrides, 2012). The mean stimulation location is shown in each hemisphere in standard MNI coordinates: (−46, 26, 30 mm) for the left 9/46v and (52, 26, 32 mm) for the right 9/46v. The stimulation site was marked and maintained using Brainsight (Rogue Research). The TMS coil position was tracked using a three-dimensional optical system (Polaris System, Northern Digital).

### Stimulation protocol

The theta-burst magnetic stimulation magnitude was based on the resting motor threshold (RMT) in primary motor cortex. The position at which left or right motor cortex was maximally excitable in eliciting motor-evoked potentials (MEPs) in the contralateral FDI muscle was determined, using single-pulse TMS (Magstim200 stimulator). The coil was placed tangentially on the scalp with the handle pointing backward and laterally at a 45° angle away from the midline. The EMG response of the FDI muscle was recorded using Ag-AgCl surface electrodes. The RMT was defined as the minimum intensity required to elicit at least 5 MEPs (>50 mV peak-to-peak amplitude) in 10 consecutive single-pulse stimulations.

cTBS (Goldsworthy et al., 2012) was used to disrupt neural activity in left or right 9/46v before learning. cTBS was applied in two trains (10 min apart) of repetitive biphasic magnetic pulses (Magstim Super Rapid Stimulator) at 70% intensity of the RMT for the FDI muscle (based on left and right M1 separately, recorded using a Magstim 200 monophasic stimulator). Each train of cTBS comprised 600 pulses applied in bursts of three pulses at 50 Hz, with bursts repeated at a frequency of 5 Hz, corresponding to a total train length of 40 s. cTBS stimulation was delivered with the coil handle pointed downward.

To test for possible indirect effects of cTBS on motor cortex, we applied single-pulse TMS to the motor hotspot, at an intensity sufficient to evoke 20 MEPs of ∼500–1000 μV (peak-to-peak amplitude) both before stimulation and at the same intensity, 10 min after cTBS.

### Statistical analysis

Directional error was quantified as the angular deviation (*AD*) from the true target direction (center of the target zone) at the maximum velocity. The absolute angular deviation, |*AD*|, was used as a measure of movement accuracy. The number of trials with positive feedback and absolute angular deviation were used to quantify learning. The rate of learning was computed through a linear fit to the absolute angular deviation as a function of learning trials. The slope of the fitted line was used as a measure of the learning rate. One-way ANOVA was performed on learning rates across experimental conditions. One-way ANOVA was also performed on mean change in absolute angular deviation from the first block to the last block of training.

We also computed a linear fit to the mean percentage of rewarded movements across participants over the course of training. One-way ANOVA was performed on changes in the percent of rewarded movements between the first and last block of training. A two-way ANOVA was performed to assess the effect of reward history on movement variability from the *n*th to *n* + 1th trial in different experimental conditions. *Post hoc* tests were corrected for multiple comparisons.

To evaluate possible effects of cTBS on motor cortex, MEPs recorded post-cTBS were expressed as a percentage of pre-cTBS MEPs, using mean MEP amplitude on a per subject basis. One-way ANOVA was used to test for the difference between experimental conditions.

## Results

**Figure 2.**
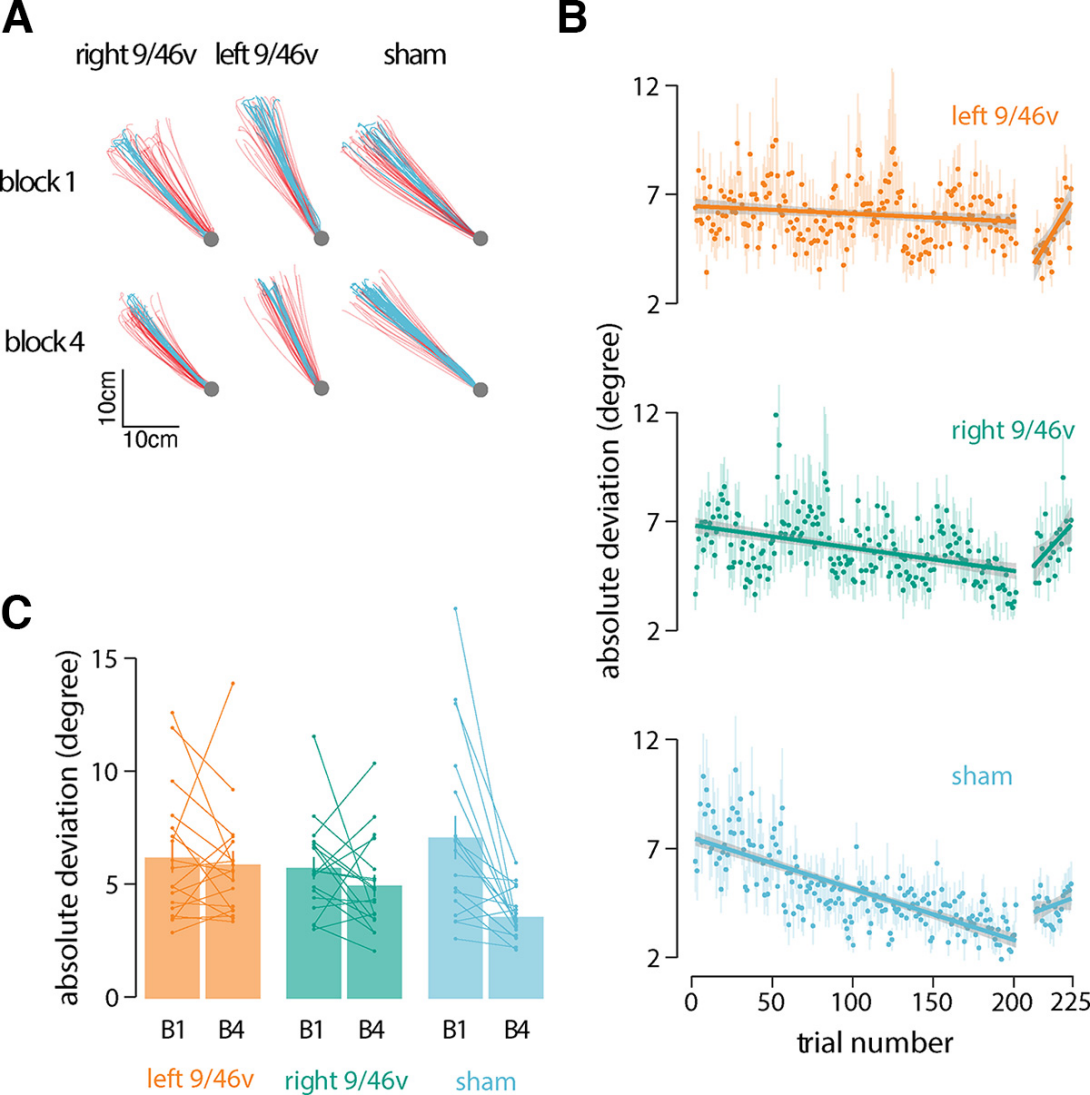
Suppression of left 9/46v using cTBS disrupts motor learning. ***A***, Hand paths of a representative participant from each group at the start (block 1) and end of training (block 4). Hand paths shown in red are for unsuccessful movements, and those in blue are for successful movements. ***B***, Mean absolute deviation from the center of the target zone over the course of training. The linear fit is shown across learning trials and no-feedback trials separately. The shaded region represents ±SEM. The rate of learning was less in participants who received stimulation over left 9/46v than those who received sham stimulation. ***C***, Mean absolute deviation in the first and last block of the training. Participants in the sham stimulation condition showed a greater reduction in |AD| than participants in the left 9/46v condition.

Participants held the handle of a robotic manipulandum (Fig. 1*A*) and made reaching movements toward a hidden target (Fig. 1*B*, shaded gray area) in four blocks of 50 trials each. Participants were rewarded for successful movement in the target direction. To assess the contribution of the ventrolateral prefrontal cortex to motor learning, cTBS stimulation was applied before learning in different groups of subjects in each hemisphere separately. Figure 2*A* shows data from a representative subject in each experimental condition. Movement paths shown in blue are for successful (rewarded) movements and those in red are for unsuccessful movements. Note that the overall direction differs in the three conditions because of individual differences in target location. The figure shows that movement paths were similar in the three experimental conditions at the beginning of training (block 1). At the end of training (block 4), participants in the sham condition moved more consistently to the target than participants who received stimulation to either left or right 9/46v (Fig. 2*A*).

Reduction in the angular deviation from the target direction, |AD|, over the course of the motor task provides a measure of improvement in accuracy as a result of learning (Fig. 2*B*). The rate of reduction in |AD| was estimated for each subject separately. ANOVA applied to the slope estimates indicated the rate of angular deviation reduction differed significantly among stimulation conditions (*F*_(2,51)_ = 4.19, *p* = 0.02; Fig. 2*B*). The rate of learning was slower in participants who received stimulation over left 9/46v (slope = −0.002, 95% CI = −0.01, 0.006) than those who received sham (slope = −0.022, 95% CI = −0.033, −0.011) stimulation (*p* = 0.016). There was no significant difference in the learning rate between the sham and right 9/46v (slope = −0.009, 95% CI = −0.019, 0.001) conditions (*p* = 0.16). Another indicator of learning is the change in the |AD| from the beginning of the learning session to the |AD| at the end (Fig. 2*C*). The mean change in |AD| from the first to last learning block showed significant differences between conditions (*F*_(2,51)_ = 4.93, *p* = 0.01). *Post hoc* tests indicated that participants in the sham stimulation condition showed a greater reduction in |AD| than participants in the left 9/46v condition (*p* = 0.014).

Participants also performed no-feedback trials after the initial learning session in which feedback on movement success was withheld. We found no significant difference in |AD| between conditions (*F*_(2,51)_ = 0.57, *p* = 0.56) nor was there a significant difference in the slope between the groups in no-feedback trials (*F*_(2,51)_ = 1.82, *p* = 0.17). The slope in these trials for the sham condition was not reliably different from zero (*p* = 0.56). The slopes in the left 9/46v (*p* = 0.003) and right 9/46v (*p* = 0.05) conditions were both found to be reliably greater than zero indicating a progressive reduction in accuracy for the learned target direction.

**Figure 3.**
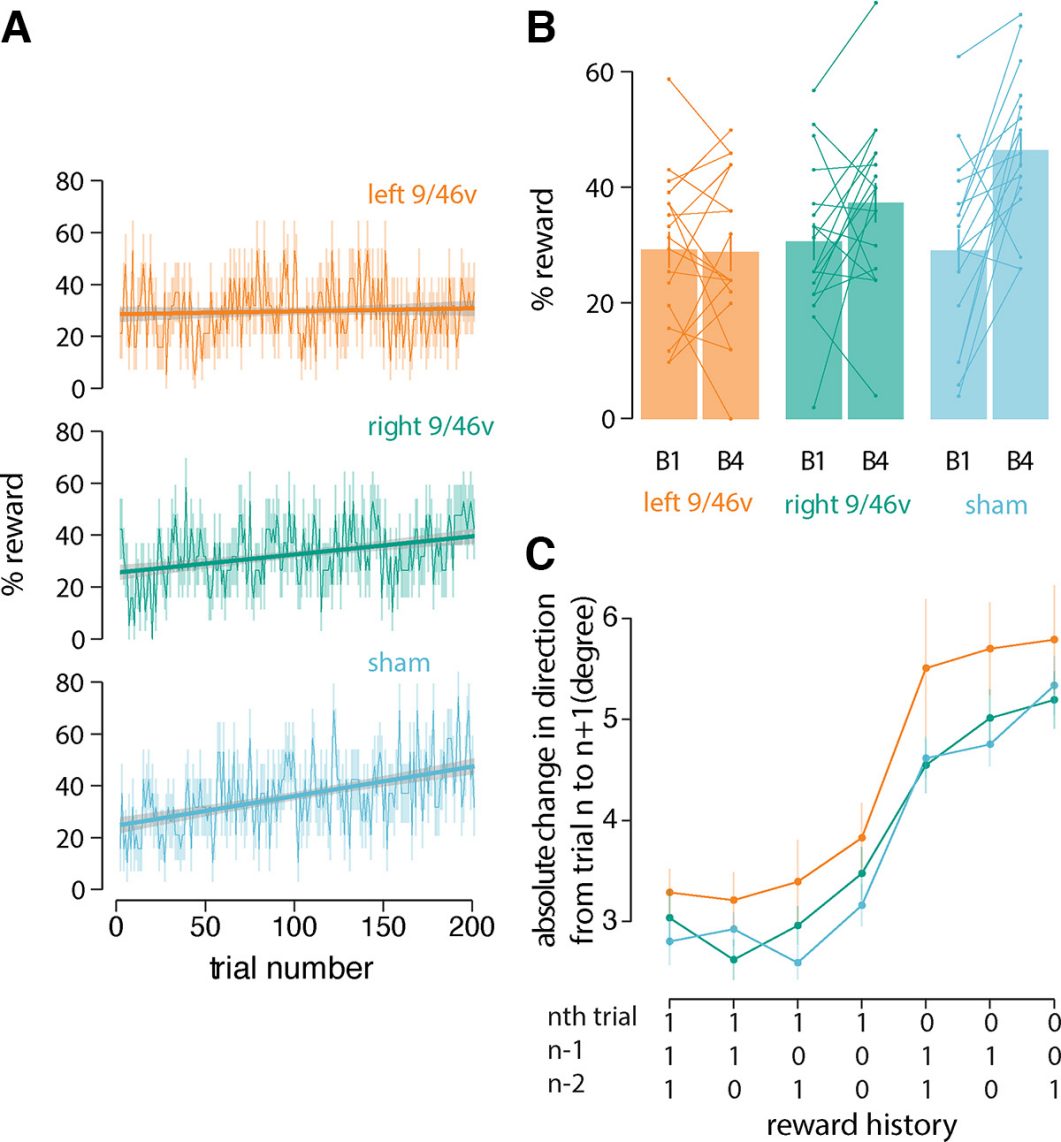
Suppression of left 9/46v using cTBS leaves reinforcement learning intact. ***A***, Mean percentage of rewarded trials over the course of training. A linear fit is shown across learning trials. The shaded region represents ±SEM. ***B***, Mean percent of rewarded movements in the first and last block of the training. Participants in the sham stimulation condition received more rewards as learning progressed, whereas participants who received stimulation to left 9/46v showed no improvement at all. ***C***, Mean absolute change in movement direction between the current trial (*n*th trial) and the subsequent trial (*n* + 1th trial) as a function of the history of rewarded movements. Reward history included three most recent movements (*n*, *n*–1, and *n*–2 trial), where at least one of these movements was rewarded. The left 9/46v group showed the same basic reward-history-dependent pattern as the other conditions but with greater change in direction overall. This suggests that the learning deficit after left 9/46v suppression is not because of inability to process reward but likely because of a deficit in memory for target direction.

During the motor learning task, participants were instructed to maximize the number of rewarded trials. Figure 3*A* shows the percentage of rewarded trials over the course of learning. Participants in the sham stimulation group showed a steady increase in the number of successful movements (slope = 0.119, 95% CI = 0.091–0.146) compared with participants in the left 9/46v stimulation condition (slope = 0.011, 95% CI = −0.014–0.038). Participants in the right 9/46v condition showed values intermediate between those in the other two conditions (slope = 0.073 95% CI = 0.047–0.098). Statistical tests were conducted to assess changes in the percent of rewarded (Figure 3*B*) movements between the first and the last block of training. The change scores (increase from start to end of training in the percent of rewarded trials) differed significantly across conditions (*F*_(2,51)_ = 6.18, *p* = 0.003). *Post hoc* tests indicated a reliable difference in reward change scores between the left 9/46v and sham stimulation conditions (*p* = 0.002). Specifically, participants in the sham stimulation condition received more rewards as learning progressed, whereas participants who received stimulation to left 9/46v showed no improvement at all. There was no difference in reward change scores for participants in the right 9/46v and sham stimulation conditions (*p* = 0.10). One sample *t* tests indicated that the reward change from the first to last block for participants in the left 9/46v condition was not reliably different from zero (*t*_(17)_ = −0.13, *p* = 0.89).

One possible reason for not showing improvement over the course of training in the left 9/46v condition was that stimulation impaired the capacity to benefit from reward. To assess this possibility, we computed the absolute change in movement direction between the current trial (nth trial) and the subsequent trial (*n* + 1th trial) as a function of the history of rewarded movements. The analysis, shown in Figure 3*C*, was conducted over the three most recent movements (*n*, *n*–1, and *n*–2 trial), under conditions where at least one of these movements was rewarded. It can be seen that there is a graded pattern of absolute change in movement direction, which is least following three rewarded movements and greatest when only a single movement is rewarded. Thus, a normal although more variable reward-history-dependent pattern is obtained following cTBS to left 9/46v. A two-way ANOVA with reward history and the stimulation condition as the independent factors and Δ*m*, the absolute change in movement direction, as dependent variable revealed a significant effect of reward history (*F*_(6,306)_ = 53.85, *p* < 0.001) indicating that change in movement direction is dependent on the number of rewarded trials in the recent past. The overall magnitude of the change in direction, Δ*m*, marginally differed across stimulation conditions (*F*_(2,51)_ = 2.86, *p* = 0.06). Bonferroni-holm corrected *post hoc* tests indicated that participants in the left 9/46v stimulation condition showed greater change in direction than participants in the sham (*p* = 0.007) and right 9/46v conditions (*p* = 0.02; Fig. 3*C*). There was no indication that the reward-history dependent pattern differed between conditions, that is, there was no significant interaction between stimulation conditions and reward history (*F*_(12,306)_ = 0.43, *p* = 0.94). In summary, participants in the left 9/46v group showed the same basic reward-history dependent pattern as the other conditions but with greater change in direction overall. This suggests that the learning deficit in the left 9/46v condition is not because of an inability to benefit from reward per se.

**Figure 4.**
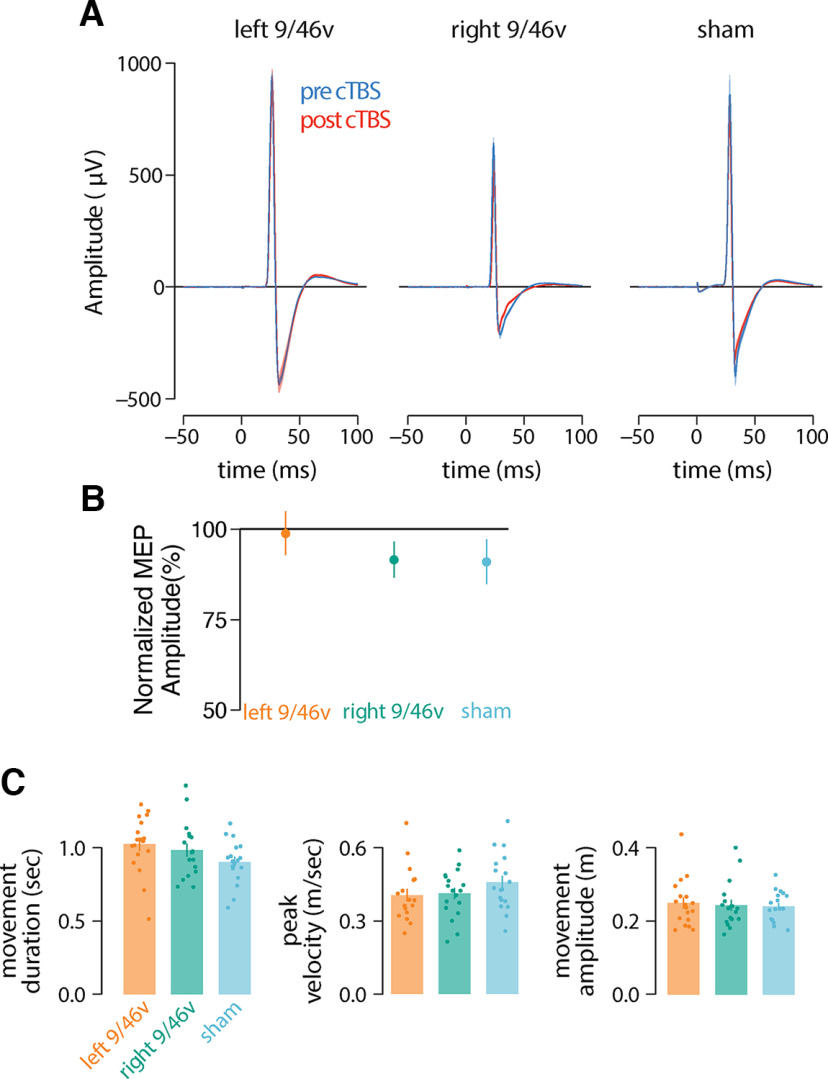
cTBS over left or right 9/46v did not alter the excitability of motor cortex or basic movement parameters. ***A***, Mean time series of MEPs recorded from the FDI muscle pre-cTBS (blue) and post-cTBS (red) from a representative participant in each experimental condition. The TMS pulse occurs at time = 0 ms. The shaded regions are ±SEM across 20 MEPs. ***B***, Mean change in amplitude of MEPs measured 10 min after cTBS (computed as a percentage of pre-cTBS MEPs). Error bars give the SE across participants. ***C***, Mean movement duration, peak velocity, and movement amplitude across experimental conditions. cTBS to either left or right 9/46v did not modify the movement parameters.

We have also assessed the possibility that 9/46v stimulation affected the movements themselves. We compared three basic movement parameters, peak velocity, movement amplitude and movement duration across stimulation conditions (Fig. 4*C*). There were no significant differences between conditions (left and right 9/46v and sham condition) in peak velocity (*F*_(2,51)_ = 1.28, *p* = 0.28), movement amplitude (*F*_(2,51)_ = 0.10, *p* = 0.90) and movement duration (*F*_(2,51)_ = 2.12, *p* = 0.13). We also tested the possibility that 9/46v stimulation indirectly affected primary motor cortex and that deficits in learning occurred as a consequence. We assessed MEPs before and after stimulation (representative sample in Fig. 4*A*) and found that there were no significant differences in peak-to-peak MEP amplitude across experimental conditions (*F*_(2,51)_ = 0.56, *p* = 0.57; Fig. 4*B*). Overall, this suggests that cTBS to 9/46v did not alter basic movement patterns nor did it indirectly act on primary motor cortex.

## Discussion

The present study used transcranial magnetic stimulation to disrupt activity in ventrolateral prefrontal cortex (9/46v) to test its involvement in human motor learning. Participants held the handle of a robot arm and made movements to a hidden target. Positive reinforcement was provided when the movement ended in the target zone. cTBS stimulation was delivered before learning either to left or right 9/46v; control participants received sham TBS. It was found that cTBS to left 9/46v all but eliminated improvements in movement as measured by changes in angular direction relative to the target. cTBS also led to a significant reduction in the number of reinforced trials in comparison to sham stimulation. The disruption of 9/46v did not adversely affect the ability to use reward as indicated by a normal, although more variable, dependence of movement direction on reward-history. As there is no visual feedback whatsoever in this task, this latter observation suggests that while a sensitivity to reward is preserved following disruption of 9/46v, there is an across-the-board deficit in somatic memory for target location or target directed movement, a result consistent with previous demonstrations of 9/46v involvement in somatic memory (Romo et al., 1999). Overall, the present results indicate that area 9/46 is part of a network that participates in human motor learning.

No feedback trials at the end of training are consistent with this conclusion. For both left and right 9/46v there is a progressive increase during no-feedback trials in angular deviation relative to the target which is suggestive of a progressive loss of information during retention testing. In contrast, the slope is not different from zero following sham stimulation indicating that retention is unimpaired when 9/46v is intact. It should be noted that while for left 9/46v stimulation retention performance appears to be initially better than that observed during learning, the values at the start of the retention test are wholly with the range of those obtained over the course of training.

Although it is in prefrontal cortex, several studies have shown that, in nonhuman primates, area 9/46v has both inputs and outputs to somatic regions of the brain, including connections to second somatosensory cortex, cortical areas PF and PFG (supramarginal gyrus) in the inferior parietal lobe (Preuss and Goldman‐Rakic, 1989; Petrides and Pandya, 2002; Gerbella et al., 2013) and also to ventral premotor cortex (Dum and Strick, 2005). In humans, an analogous pattern of connectivity between this same set of areas has been reported using resting-state fMRI and diffusion tractography (Barbeau et al., 2020) and between the ventral portion of the middle frontal gyrus and ventral premotor cortex (Catani et al., 2012). Outputs from the basal ganglia to area 9/46v have also been reported (Middleton and Strick, 2002). As such, this area is well placed to funnel both somatic (error-based) and reinforcement-based information to frontal motor areas in support of learning. The involvement of area 9/46v in somatic memory and decision-making has been documented in studies in which nonhuman primates are required to hold in memory vibrotactile information (delivered to the fingertips) and to make judgements regarding relative frequency. Neurons in this region have been found to show both memory dependent and decision-making related activity (Romo et al., 1999).

Studies of spatial working memory in humans also report activity in this same region of prefrontal cortex (D’Esposito et al., 1998; Owen et al., 2005). In strictly behavioral studies, there is evidence of a relationship between somatosensory memory and reinforcement learning (Sidarta et al., 2018) and also between visuospatial memory and sequence learning (Bo and Seidler, 2009; Bo et al., 2009). In the study by Sidarta and colleagues (Sidarta et al., 2018), using a task similar to the one in the present study, it was found that individuals with better sensory memory for their own movements also showed greater learning.

Reinforcement learning has been characterized as involving both repetition of successful movements (exploitation) or the selection of new movements following unsuccessful trials (exploration). The present results suggest that disruption of 9/46v leaves both processes intact as indicated by the finding that a normal, but more variable, dependence of movement on reward history is preserved. The deficits in learning appear instead to be memory dependent. This finding shows that it is possible experimentally to partially dissociate the contribution of brain structures involved in reward and sensory memory in motor learning. Area 9/46v involvement in human motor learning has been reported in studies involving both reinforcement and error-based learning where learning-related activity is observed in both task-based and resting-state scans (Anguera et al., 2010; Sidarta et al., 2016).

It was found that disruption of activity in right 9/46v resulted in a reduction in both the rate of learning and the number of reinforced trials. Although these effects were not statistically different from measures of the same variables when stimulation was delivered to left 9/46v, nor when sham stimulation was delivered, the results for right hemisphere stimulation are intermediate between the two. Activity in right 9/46v has been observed previously in humans in both reinforcement learning and error-based learning tasks (Anguera et al., 2010; Sidarta et al., 2016). It has also been observed previously in sensory memory tasks in nonhuman primates (Romo et al., 1999). The extent to which there is hemispheric specialization in the contribution of area 9/46v to learning is uncertain. In humans, there is substantial interhemispheric connectivity in prefrontal cortex (Zarei et al., 2006). Moreover, interhemispheric propagation of TMS stimulation in prefrontal cortex has been reported (Voineskos et al., 2010), which makes possible the idea that the partial disruption of learning which occurs when right 9/46v is stimulated occurs as a result of indirect effects on the left hemisphere.

The involvement of a somatic network in human motor learning is supported by the finding that areas which show somatic memory and decision-making activity in nonhuman primates, second somatosensory cortex, ventral premotor cortex, supplementary motor area, and ventrolateral prefrontal cortex (Romo et al., 2012) are likewise areas that show learning-related changes in functional connectivity following motor learning in humans (Vahdat et al., 2011). This somatic network which also includes inferior parietal cortex (supramarginal and angular gyrus; Barbeau et al., 2020) fits within a broader interconnected sensorimotor network which includes primary motor and somatosensory cortex, medial wall motor areas, the superior parietal lobule, basal ganglia, and cerebellum (for review, see Rizzolatti and Luppino, 2001; Bostan and Strick, 2018). While each of these areas might contribute to the learning observed in the present study, the elimination of learning following cTBS to 9/46v suggests a causal contribution of this specific area in the context of reinforcement motor learning in humans.

In summary, it was found that cTBS stimulation to area 9/46v in prefrontal cortex disrupts motor learning without affecting the movements themselves. The deficit appears to be primarily related to impaired somatic memory for target location or target directed movement; disruption of 9/46v leaves reinforcement-based learning largely intact. 9/46v is distinguished from other regions of prefrontal cortex by its significant pattern of somatosensory connectivity. Area 9/46v thus appears to be part of the human motor learning circuit.

## Synthesis

Reviewing Editor: David Franklin, Technische Universitat Munchen Decisions are customarily a result of the Reviewing Editor and the peer reviewers coming together and discussing their recommendations until a consensus is reached. When revisions are invited, a fact-based synthesis statement explaining their decision and outlining what is needed to prepare a revision will be listed below. The following reviewer(s) agreed to reveal their identity: Ronan Mooney.

Your manuscript has been reviewed by two experts in the field. They both have interest in the manuscript and think that this is important work providing further evidence of the role of the ventrolateral prefrontal cortex in reward-based motor learning. However, we have concerns with some of the technical aspects and instructions to the subjects, and would like further discussion on the results of the retention phase. Finally, we think that the manuscript would benefit from a more comprehensive comparison with past work in the literature, with a specific focus on the anatomical and physiological evidence that also supports this work. I encourage you to revise your manuscript, taking into account each of the concerns raised by the reviewers, and preparing a point-point-response.

General Comments from the reviewers.

The study assessed the effects of cTBS over ventrolateral prefrontal cortex on reinforcement-based motor learning. The data provide further evidence for the role of this region in reward-based motor learning, complimenting imaging and non-human primate studies. Overall, the study is interesting, however the manuscript has some shortcomings.

What were the participants instructed to do during the 25 post-training trials with no feedback (e.g., continue reaching in the direction which produced reward during training)? The instructions could have a major impact on the observed data.

Was the MagStim Rapid stimulator used to determine RMT, or was the RMT value used to set the cTBS intensity based off of the MagStim200 stimulator? The MagStim Rapid delivers a biphasic pulse, whereas the MagStim 200 is monophasic and can therefore result in differences in RMT within an individual.

The learning slope (angular deviation change) was shallower in the left 46v group compared to sham. However, the lack of difference in performance during the retention block is perplexing. In fact, the left 46v group looks to be performing slightly better during the beginning of the retention period. How do the authors interpret these results in the context of the disruption with cTBS?

I agree that the ventrolateral prefrontal cortex might be involved in motor learning. Anatomic and neurophysiological data support this hypothesis. However, it is important that the authors sustain this claim based in previous literature and current results. A) I think that the authors must re-review anatomic literature. As far as I know, Giacomo Rizzolatti reviewed the cortical motor network and the authors should check this. B) Concerning the neurophysiological data, Romo et al. have shown that not only the prefrontal cortex contains somatic working memory fields but also premotor fields of frontal cortex and second somatosensory cortex as well. Therefore, practically all these fields are connected and each is of these cortical nodes might contribute motor learning shown here. Authors must review all this literature.

The authors should be very careful with the terminology used here: they jump from ventrolateral, middle, BA9/46 along the text. They should use one, or in the methods section more specific: BA9/46.

Minor points

The target MEP amplitude to determine cTBS effects on motor cortex excitability is likely meant to be in microvolts not millivolts.

Please describe how the MEP amplitude data were analyzed and which statistical tests were carried out.

## References

[B1] Anguera JA, Reuter-Lorenz PA, Willingham DT, Seidler RD (2010) Contributions of spatial working memory to visuomotor learning. J Cogn Neurosci 22:1917–1930. 10.1162/jocn.2009.21351 19803691

[B2] Barbas H, Pandya DN (1989) Architecture and intrinsic connections of the prefrontal cortex in the rhesus monkey. J Comp Neurol 286:353–375. 10.1002/cne.902860306 2768563

[B3] Barbeau EB, Descoteaux M, Petrides M (2020) Dissociating the white matter tracts connecting the temporo-parietal cortical region with frontal cortex using diffusion tractography. Sci Rep 10:8186. 10.1038/s41598-020-64124-y 32424290 PMC7235086

[B4] Bernardi NF, Darainy M, Ostry DJ (2015) Somatosensory contribution to the initial stages of human motor learning. J Neurosci 35:14316–14326. 10.1523/JNEUROSCI.1344-15.2015 26490869 PMC4683690

[B5] Bo J, Seidler RD (2009) Visuospatial working memory capacity predicts the organization of acquired explicit motor sequences. J Neurophysiol 101:3116–3125. 10.1152/jn.00006.2009 19357338 PMC2694099

[B6] Bo J, Borza V, Seidler RD (2009) Age-related declines in visuospatial working memory correlate with deficits in explicit motor sequence learning. J Neurophysiol 102:2744–2754. 10.1152/jn.00393.2009 19726728 PMC2777814

[B7] Bostan AC, Strick PL (2018) The basal ganglia and the cerebellum: nodes in an integrated network. Nat Rev Neurosci 19:338–350. 10.1038/s41583-018-0002-7 29643480 PMC6503669

[B8] Catani M, Dell’Acqua F, Vergani F, Malik F, Hodge H, Roy P, Valabregue R, de Schotten MT (2012) Short frontal lobe connections of the human brain. Cortex 48:273–291. 10.1016/j.cortex.2011.12.001 22209688

[B9] Codol O, Holland PJ, Manohar SG, Galea JM (2020) Reward-based improvements in motor control are driven by multiple error-reducing mechanisms. J Neurosci 40:3604–3620. 10.1523/JNEUROSCI.2646-19.2020 32234779 PMC7189755

[B10] Dayan E, Herszage J, Laor‐Maayany R, Sharon H, Censor N (2018) Neuromodulation of reinforced skill learning reveals the causal function of prefrontal cortex. Hum Brain Mapp 39:4724–4732. 10.1002/hbm.24317 30043536 PMC6866473

[B11] D’Esposito M, Aguirre GK, Zarahn E, Ballard D, Shin RK, Lease J (1998) Functional MRI studies of spatial and nonspatial working memory. Cogn Brain Res 7:1–13. 10.1016/S0926-6410(98)00004-4 9714705

[B12] Dum RP, Strick PL (2005) Frontal lobe inputs to the digit representations of the motor areas on the lateral surface of the hemisphere. J Neurosci 25:1375–1386. 10.1523/JNEUROSCI.3902-04.2005 15703391 PMC6726000

[B13] Fermin ASR, Yoshida T, Yoshimoto J, Ito M, Tanaka SC, Doya K (2016) Model-based action planning involves cortico-cerebellar and basal ganglia networks. Sci Rep 6:31378. 10.1038/srep31378 27539554 PMC4990901

[B14] Frankle WG, Laruelle M, Haber SN (2006) Prefrontal cortical projections to the midbrain in primates: evidence for a sparse connection. Neuropsychopharmacology 31:1627–1636. 10.1038/sj.npp.1300990 16395309

[B15] Gerbella M, Borra E, Tonelli S, Rozzi S, Luppino G (2013) Connectional heterogeneity of the ventral part of the macaque area 46. Cereb Cortex 23:967–987. 10.1093/cercor/bhs096 22499799

[B16] Goldsworthy MR, Pitcher JB, Ridding MC (2012) The application of spaced theta burst protocols induces long-lasting neuroplastic changes in the human motor cortex: the application of spaced theta burst protocols. Eur J Neurosci 35:125–134. 10.1111/j.1460-9568.2011.07924.x 22118241

[B17] Hikosaka K, Watanabe M (2000) Delay activity of orbital and lateral prefrontal neurons of the monkey varying with different rewards. Cereb Cortex 10:263–271. 10.1093/cercor/10.3.263 10731221

[B18] Middleton FA, Strick PL (2002) Basal-ganglia ‘projections’ to the prefrontal cortex of the primate. Cereb Cortex 12:926–935. 10.1093/cercor/12.9.926 12183392

[B19] Miller EK, Cohen JD (2001) An integrative theory of prefrontal cortex function. Annu Rev Neurosci 24:167–202. 10.1146/annurev.neuro.24.1.167 11283309

[B20] Oldfield RC (1971) The assessment and analysis of handedness: the Edinburgh inventory. Neuropsychologia 9:97–113. 10.1016/0028-3932(71)90067-4 5146491

[B21] Owen AM, McMillan KM, Laird AR, Bullmore E (2005) N‐back working memory paradigm: a meta‐analysis of normative functional neuroimaging studies. Hum Brain Mapp 25:46–59. 10.1002/hbm.20131 15846822 PMC6871745

[B22] Petrides M (2012) The human cerebral cortex: an MRI atlas of the sulci and gyri in MNI stereotaxic space. San Diego: Elsevier/Academic Press.

[B23] Petrides M, Pandya DN (2002) Comparative cytoarchitectonic analysis of the human and the macaque ventrolateral prefrontal cortex and corticocortical connection patterns in the monkey. Eur J Neurosci 16:291–310. 10.1046/j.1460-9568.2001.02090.x 12169111

[B24] Preuss TM, Goldman‐Rakic PS (1989) Connections of the ventral granular frontal cortex of macaques with perisylvian premotor and somatosensory areas: anatomical evidence for somatic representation in primate frontal association cortex. J Comp Neurol 282:293–316. 10.1002/cne.902820210 2708598

[B25] Rizzolatti G, Luppino G (2001) The cortical motor system. Neuron 31:889–901. 10.1016/s0896-6273(01)00423-8 11580891

[B26] Romo R, Brody CD, Hernández A, Lemus L (1999) Neuronal correlates of parametric working memory in the prefrontal cortex. Nature 399:470–473. 10.1038/20939 10365959

[B27] Romo R, Lemus L, de Lafuente V (2012) Sense, memory, and decision-making in the somatosensory cortical network. Curr Opin Neurobiol 22:914–919. 10.1016/j.conb.2012.08.002 22939031

[B28] Sidarta A, Vahdat S, Bernardi NF, Ostry DJ (2016) Somatic and reinforcement-based plasticity in the initial stages of human motor learning. J Neurosci 36:11682–11692. 10.1523/JNEUROSCI.1767-16.2016 27852776 PMC5125226

[B29] Sidarta A, van Vugt FT, Ostry DJ (2018) Somatosensory working memory in human reinforcement-based motor learning. J Neurophysiol 120:3275–3286. 10.1152/jn.00442.2018 30354856 PMC6337046

[B30] Vahdat S, Darainy M, Milner TE, Ostry DJ (2011) Functionally specific changes in resting-state sensorimotor networks after motor learning. J Neurosci 31:16907–16915. 10.1523/JNEUROSCI.2737-11.2011 22114261 PMC3260885

[B31] Voineskos AN, Farzan F, Barr MS, Lobaugh NJ, Mulsant BH, Chen R, Fitzgerald PB, Daskalakis ZJ (2010) The role of the corpus callosum in transcranial magnetic stimulation induced interhemispheric signal propagation. Biol Psychiatry 68:825–831. 10.1016/j.biopsych.2010.06.021 20708172

[B32] Wallis JD, Miller EK (2003) From rule to response: neuronal processes in the premotor and prefrontal cortex. J Neurophysiol 90:1790–1806. 10.1152/jn.00086.2003 12736235

[B33] Williams SM, Goldman-Rakic PS (1998) Widespread origin of the primate mesofrontal dopamine system. Cereb Cortex 8:321–345. 10.1093/cercor/8.4.321 9651129

[B34] Yeterian EH, Pandya DN, Tomaiuolo F, Petrides M (2012) The cortical connectivity of the prefrontal cortex in the monkey brain. Cortex 48:58–81. 10.1016/j.cortex.2011.03.004 21481342 PMC3161133

[B35] Zarei M, Johansen‐Berg H, Smith S, Ciccarelli O, Thompson AJ, Matthews PM (2006) Functional anatomy of interhemispheric cortical connections in the human brain. J Anat 209:311–320. 10.1111/j.1469-7580.2006.00615.x 16928200 PMC2100336

